# Newly Diagnosed VEXAS (Vacuoles, E1 Enzyme, X-linked, Autoinflammatory, Somatic) Syndrome in a Patient Presenting With Legionella Pneumonia

**DOI:** 10.7759/cureus.111837

**Published:** 2026-06-30

**Authors:** Alejandro Sanchez Jimenez, Christian Hirt, Sidharth Anand, Steven Tsai, Jessica Ma

**Affiliations:** 1 Department of Medicine, University of California, Los Angeles, Los Angeles, USA; 2 Department of Pathology and Laboratory Medicine, University of California, Los Angeles, Los Angeles, USA; 3 Department of Medicine, Division of Hematology-Oncology, University of California, Los Angeles, Los Angeles, USA

**Keywords:** autoimmune, bone marrow biopsy, clinical hematology, hematology-oncology, legionella pneumonia, myelodysplastic syndromes, pathology, uveitis, vexas, vexas syndrome

## Abstract

VEXAS (vacuoles, E1 enzyme, X-linked, autoinflammatory, somatic) syndrome is a newly described inflammatory disease characterized by recurrent autoimmune manifestations, such as chondritis and uveitis, in the setting of classic laboratory abnormalities, including elevated inflammatory markers and macrocytic anemia. It is caused by an acquired mutation in the UBA1 gene within the myeloid lineage, ultimately resulting in cell death and systemic inflammation. Timely diagnosis can help expedite treatment and encourage monitoring for complications of the disease, most notably a predilection to infections.

A 77-year-old male presented to the emergency room due to new-onset fevers and shortness of breath. His history was notable for six months of non-specific symptoms such as fatigue and weight loss, as well as recurrent inflammatory syndromes, including uveitis and joint swelling. At one point, he was treated empirically with steroids due to concern for giant cell arteritis; however, biopsy did not confirm this diagnosis. Labs were notable for significantly elevated inflammatory markers and a macrocytic anemia. His hospitalization was notable for acute hypoxemic respiratory failure requiring high-flow nasal cannula oxygen. His condition improved with broad-spectrum antibiotics, and he was ultimately diagnosed with *Legionella* pneumonia when *Legionella* PCR from pleural fluid returned positive. Prior to discharge, genetic testing for the UBA1 gene mutation and bone marrow biopsy demonstrating vacuolization of myeloid precursors confirmed the diagnosis of VEXAS syndrome. He was started on prednisone and azacitidine in the outpatient setting, which led to significant improvement in symptoms, inflammatory markers, and cell counts.

## Introduction

VEXAS (vacuoles, E1 enzyme, X-linked, autoinflammatory, somatic) syndrome is a recently identified inflammatory disease characterized by immune dysregulation and progressive hematologic dysfunction. It often manifests as recurrent episodes of autoinflammation such as arthritis, uveitis, chondritis, and rashes in addition to laboratory abnormalities, most classically an unexplained macrocytic anemia and elevated inflammatory markers [1,2]. It is caused by an acquired mutation in UBA1, a gene on the X chromosome that codes for E1-ubiquitin-activating enzyme, which plays an imperative role in maintaining protein homeostasis within cells [3]. The pathophysiology behind VEXAS syndrome is thought to be driven by the accumulation of misfolded proteins within mutant myeloid cells, leading to increased inflammasome activation and cell death [1,4].

VEXAS syndrome, albeit a relatively newly discovered disease entity, is estimated to have a prevalence of about one in 4,000 in men over age 50 [5]. However, many patients remain undiagnosed, likely due to limited awareness by clinicians of this novel disorder. VEXAS syndrome can closely mimic other vasculitic or hematologic syndromes, making diagnosis difficult. In the original paper describing VEXAS syndrome, many participants met diagnostic criteria for various other conditions, including relapsing polychondritis, Sweet’s syndrome, myelodysplastic syndrome, multiple myeloma or monoclonal gammopathy of undetermined significance, polyarteritis nodosa, and giant cell arteritis [1].

This case exemplifies both the difficulty in diagnosing this obscure disease entity and the infectious complications that arise from hematologic and immune dysfunction in VEXAS patients.

## Case presentation

A 77-year-old male with a past medical history notable for type 2 diabetes mellitus, atrial fibrillation treated with apixaban, hypertension, prostate cancer status post resection, and provoked pulmonary embolism presented to the emergency room due to new-onset fevers and shortness of breath in the setting of several months of generalized malaise, recurrent ocular inflammation, and recently diagnosed renal cell carcinoma (RCC) status post nephrectomy.

History of present illness

Six months before the current presentation, the patient presented to another hospital due to acute left eye redness and swelling, headaches, and diplopia. He denied scalp tenderness or jaw pain but did note a history of night sweats, decreased exercise tolerance, weight loss, and longstanding rashes of the limbs previously diagnosed as purpura. During the hospitalization, he was also noted to have elevated inflammatory markers. Due to concern for giant cell arteritis, he received high-dose intravenous (IV) steroids and underwent a biopsy of the left temporal artery, which demonstrated mild to moderate intimal thickening but no active inflammation. He was discharged on prednisone and referred to an outpatient rheumatology clinic for further management.

During the initial rheumatology evaluation, about two weeks after being discharged, the complete blood count (CBC) was notable for thrombocytopenia and a normal hemoglobin value with macrocytic red blood cells. Testing for hepatitis C, hepatitis B, tuberculosis, C3, C4, thyroid-stimulating hormone (TSH), rheumatoid factor, and anti-Smith, ribonucleoprotein, cyclic citrullinated peptide (CCP), Sjögren's syndrome-related antigen A (SSA), and Sjögren's syndrome-related antigen B (SSB) antibodies was unremarkable. Erythrocyte sedimentation rate (ESR) and C-reactive protein (CRP) were normal, but ferritin was mildly elevated (Table 1). The patient was on prednisone 20 mg daily at the time of the evaluation with plans to taper further over the coming weeks.

**Table 1:** Laboratory evaluation at the initial rheumatology outpatient visit (six months prior to current presentation). Of note, the patient was on prednisone at the time of evaluation. Hgb = hemoglobin; MCV = mean corpuscular volume; WBC = white blood cells; AST = aspartate aminotransferase; ALT = alanine aminotransferase; ALP = alkaline phosphatase; ESR = erythrocyte sedimentation rate; CRP = C-reactive protein; CCP = cyclic citrullinated peptide; Ab = antibody; RF = rheumatoid factor; Sm = Smith; RNP = ribonucleoprotein; HCV = hepatitis C virus; HBc = hepatitis B core; HBS = Hepatitis B surface; MTB = Mycobacterium tuberculosis; ELISA = enzyme-linked immunosorbent assay; H = high; L = low; C-ANCA = cytoplasmic antineutrophil cytoplasmic antibodies; P-ANCA = perinuclear antineutrophil cytoplasmic antibodies; TSH = thyroid-stimulating hormone; ANA = antinuclear antibodies; SSA = Sjögren's syndrome-related antigen A; SSB = Sjögren's syndrome-related antigen B.

Category	Parameter	Result	Reference range
Blood	Hgb	14.2 g/dL	13.5-17.1 g/dL
	MCV	107.4 fL (H)	79.3-98.6 fL
	WBC	8.45 x 10^3^/µL	4.15-9.95 x 10^3^/µL
	Platelets	102 x 10^3^/µL (L)	143-398 x 10^3^/µL
Chemistry	Creatinine	0.85 mg/dL	0.60-1.30 mg/dL
	AST	15 U/L	13-62 U/L
	ALT	20 U/L	8-70 U/L
	ALP	78 U/L	37-133 U/L
Rheumatologic	ESR	9 mm/hr	≤12 mm/hr
	CRP	0.3 mg/dL	<0.8 mg/dL
	Ferritin	366 ng/mL (H)	8-350 ng/mL
	CCP Ab, IgG/A	3 units	0-19 units
	C-ANCA	<1:20 titer	<1:20 titer
	P-ANCA	<1:20 titer	<1:20 titer
	RF	<10 IU/mL	<14 IU/mL
	Sm Ab	<20 units	<20 units
	RNP Ab	<20 units	<20 units
	C3	117 mg/dL	86-175 mg/dL
	C4	20 mg/dL	10-40 mg/dL
	TSH	0.91 mcIU/mL	0.3-4.7 mcIU/mL
	ANA	1:80 (nucleolar) titer (H)	<1:40 titer
	SSA Ab	<20 units	<20 units
	SSB Ab	<20 units	<20 units
Infectious	HCV Antibody	Nonreactive	Nonreactive
	HBc Ab	Nonreactive	Nonreactive
	HBS Antigen	Nonreactive	Nonreactive
	MTB Quantiferon-ELISA	Negative	Negative

As part of the workup, computed tomography (CT) of the chest, abdomen, and pelvis was performed to further evaluate for evidence of vasculitis. No CT evidence of vasculitis was noted; however, a ~4 cm right-sided renal mass was incidentally found. Three months before the current presentation, he underwent a right-sided radical nephrectomy; pathology revealed clear cell RCC. Throughout this period, the patient still experienced ongoing generalized symptoms and noted gradual worsening after nephrectomy.

Two months before the current presentation, he presented to his ophthalmologist due to a “red and irritated” right eye. On slit-light examination, he was found to have marked right anterior uveitis. At the time, he was no longer on systemic steroids. Dexamethasone eye drops were initiated. One week later, he followed up with his rheumatologist where he noted ongoing eye pain, diplopia, neck stiffness, lack of stamina, decreased appetite, headaches, and ongoing weight loss.

One month before the current presentation, the patient presented to his primary care physician with swelling, erythema, and warmth overlying the left elbow and proximal forearm. Labs at the time were notable for a macrocytic anemia, thrombocytopenia, and markedly elevated inflammatory markers. Additional testing performed during this period included angiotensin-converting enzyme, rapid plasma reagin (RPR), direct antiglobulin testing, B12, lactate dehydrogenase (LDH), and TSH, which were normal. Additionally, HLA B27 was negative (Table 2). At this point, the vast majority of diseases initially on the differential diagnosis for this patient had been excluded, given the extensive yet remarkably negative workup thus far. Most vasculitis syndromes, as well as infectious etiologies, were essentially excluded based on biopsy, imaging, and lab results. Additionally, a paraneoplastic process secondary to RCC was considered, but was thought to be unlikely given the patient was status post nephrectomy without evidence of recurrence on subsequent imaging. During outpatient evaluations with hematology-oncology, hemophagocytic lymphohistiocytosis (HLH) was considered, and the patient was scheduled for an outpatient CT-guided bone marrow biopsy.

**Table 2:** Laboratory results from outpatient visits approximately one month before presentation. Hgb = hemoglobin; MCV = mean corpuscular volume; WBC = white blood cells; Cr = creatinine; ESR = erythrocyte sedimentation rate; CRP = C-reactive protein; ACE = angiotensin-converting enzyme; RPR = rapid plasma reagin; LDH = lactate dehydrogenase; DAT = direct antiglobulin test; H = high; L = low. * CRP values were converted from mg/L to mg/dL to facilitate comparison with other lab assays in this report.

Parameter	Result	Reference range
Hgb	8.5 g/dL (L)	13.2-17.1 g/dL
MCV	113.9 fL (H)	81.4-101.7 fL
WBC	7.6 x 10^3^/µL	3.8-10.8 x 10^3^/µL
Platelets	122 x 10^3^/µL (L)	140-400 x 10^3^/µL
Creatinine	1.29 mg/dL (H)	0.70-1.28 mg/dL
ESR	128 mm/hr (H)	≤20 mm/hr
CRP	11.7 mg/dL (H)*	<0.8 mg/dL*
HLA-B27	Negative	N/A
ACE	24 U/L	9-67 U/L
RPR	Nonreactive	Nonreactive
B12	614 pg/mL	254-1060 pg/mL
LDH	204 U/L	125-256 U/L
DAT	Negative	Negative

Hospital course

On the day of admission, the patient initially presented to an urgent care clinic due to a new fever of 102° Fahrenheit (38.9°C). Due to concern for infection, he was referred to the emergency department. In the emergency room, temperature reached 101.5° Fahrenheit (38.6°C), heart rate was 80-90 bpm, and blood pressure remained stable. Labs on the day of admission were notable for a markedly elevated ferritin, ESR, and CRP, as well as a macrocytic anemia, thrombocytopenia, and normal white blood cell count (Table 3). Chest X-ray was notable for a right lower lobe consolidation concerning for a developing pneumonia given the clinical presentation (Figure 1). He was started on ceftriaxone and azithromycin for treatment of community-acquired pneumonia and admitted to the hospital for further management.

**Table 3:** Laboratory results from the day of admission. Hgb = hemoglobin; MCV = mean corpuscular volume; WBC = white blood cells; AST = aspartate aminotransferase; ALT = alanine aminotransferase; ESR = erythrocyte sedimentation rate; CRP = C-reactive protein; H = high; L = low.

Parameter	Result	Reference range
Hgb	8.5 g/dL (L)	13.7-17.5 g/dL
MCV	112.9 fL (H)	79-94.8 fL
WBC	7.54 x 10^3^/µL	3.98-10.04 x 10^3^/µL
Platelets	91 x 10^3^/µL (L)	163-369 x 10^3^/µL
Creatinine	1.39 mg/dL (H)	0.60-1.30 mg/dL
AST	43 U/L	13-62 U/L
ALT	37 U/L	8-70 U/L
ESR	74 mm/hr (H)	≤12 mm/hr
CRP	14.9 mg/dL (H)	<0.8 mg/dL
Ferritin	1533 ng/mL (H)	8-350 ng/mL

**Figure 1:**
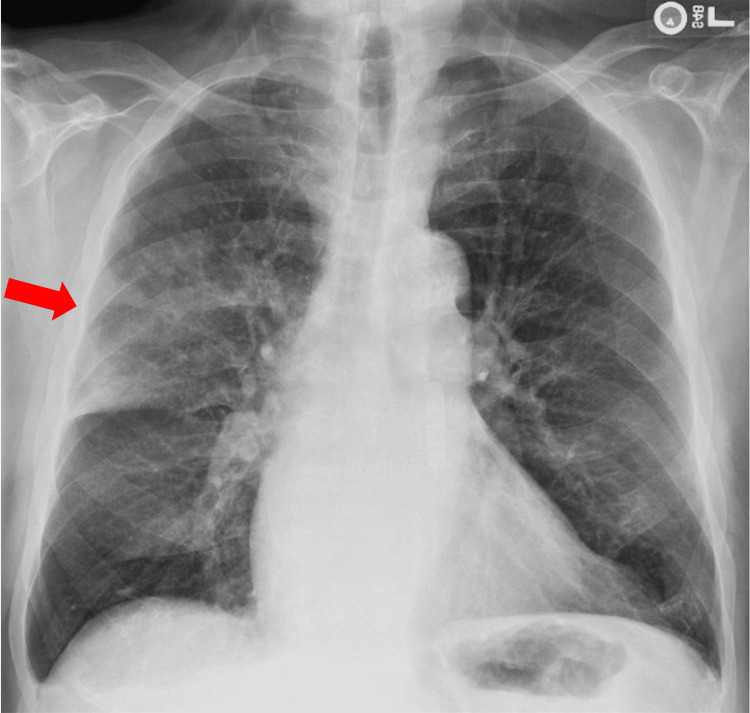
Chest X-ray from the day of admission was notable for mildly hyperinflated lungs, new consolidation within the superior aspect of the right lower lobe (red arrow), and mild bronchial wall thickening.

His hospital course was complicated by worsening acute hypoxic respiratory failure, ultimately requiring high-flow nasal cannula oxygen up to 60% fraction of inspired oxygen (FiO_^2^_) at 40 liters per minute. Due to these increasing oxygen requirements, a chest X-ray was repeated on hospital day three, which was concerning for worsening pneumonia (Figure 2). Antibiotics were broadened to vancomycin and meropenem, with azithromycin continued for a total of three days. On hospital day five, given ongoing oxygen requirements, a CT chest angiogram with contrast was performed, which was notable for a left upper lobe subsegmental pulmonary embolus, large right upper lobe consolidation with right-sided lymphadenopathy concerning for pneumonia, scattered centrilobular ground-glass opacities throughout the left lung suggestive of an infectious process, and new moderate-sized right-sided pleural effusion (Figure 3). At this point, the patient was already on therapeutic anticoagulation with IV heparin for his atrial fibrillation; however, therapy with apixaban was briefly interrupted previously in anticipation of a bone marrow biopsy. The patient's blood cultures, sputum cultures, *Legionella* urinary antigen, cryptococcal antigen, *Coccidioides* IgG and IgM, acid-fast stain, *methicillin-resistant Staphylococcus aureus* (MRSA) nares, and HIV antigen/antibody 4th-generation testing were all negative.

**Figure 2:**
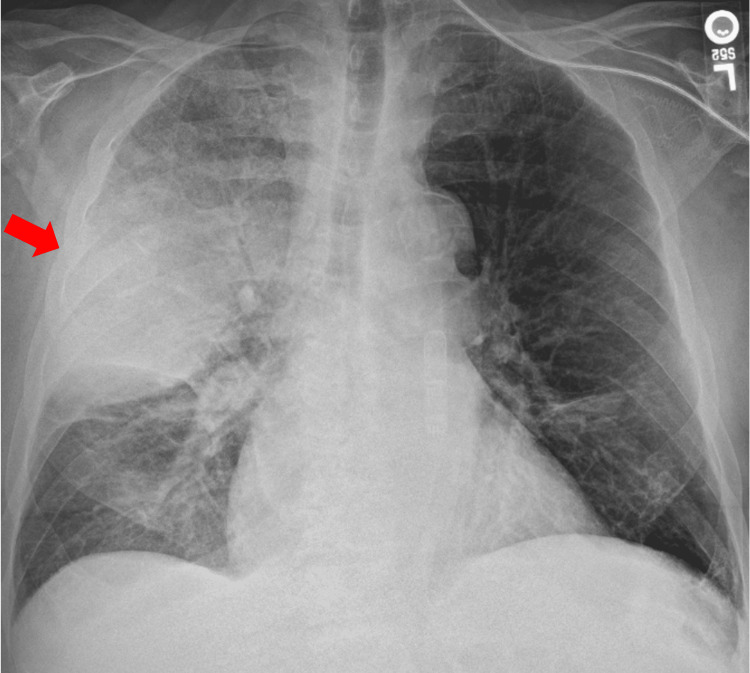
Chest X-ray from hospital day three was notable for worsening right-sided pneumonia (red arrow).

**Figure 3:**
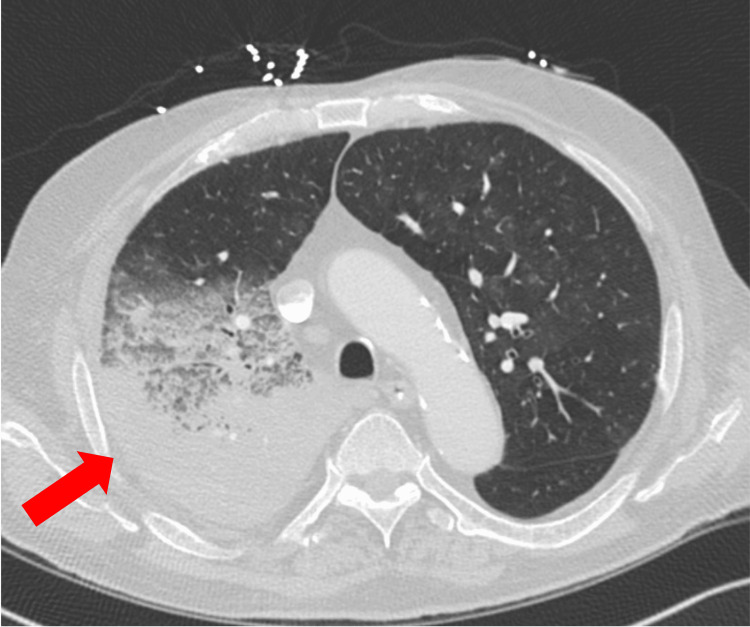
CT chest angiogram from hospital day five was notable for large right upper lobe consolidation (red arrow), likely lobar pneumonia, scattered centrilobular ground-glass opacities throughout the left lung, and a new moderate-sized right-sided pleural effusion. Not visualized here is left upper lobe subsegmental pulmonary embolus, also noted on CT scan.

On hospital day six, he underwent thoracentesis of the right pleural effusion with 1.1 liters of fluid removed. Pleural fluid was exudative based on Light’s criteria and neutrophilic predominant (Table 4). Acid-fast and Gram staining were both negative, and fluid analysis was negative for malignant cells. The patient’s respiratory status gradually improved toward the end of his hospitalization despite narrowing antibiotics to cefepime. By hospital day eight, he was breathing comfortably on room air, and on hospital day nine, he was discharged. Given clinical improvement in respiratory status and fevers following thoracentesis of the likely parapneumonic pleural effusion, the patient was recommended to complete a four-week course of oral amoxicillin-clavulanate from the day of thoracentesis with plans for outpatient infectious disease follow-up and repeat imaging.

**Table 4:** Results of pleural fluid analysis and associated serum values. LDH = lactate dehydrogenase; RBC = red blood cells.

Category	Parameter	Pleural fluid	Serum	Reference range (serum)
Chemistry	LDH	288 U/L	315 U/L	125-256 U/L
	Protein	2.9 g/dL	6.3 g/dL	6.1-8.2 g/dL
	Glucose	210 mg/dL	186 mg/dL	65-99 mg/dL
Cell counts and differential	RBC count	2,000	-	-
	Total nucleated cells	1717	-	-
	Neutrophil	52%	-	-
	Lymphocyte	30%	-	-
	Plasma cell	2%	-	-
	Monocyte	11%	-	-
	Mesothelial	5%	-	-

Prior to discharge, the patient underwent further testing for his six-month history of recurrent inflammation and cytopenias. Additionally, there was a new concern for immune dysfunction given the severity of his pneumonia without an appropriate white blood cell response. Overall, there was high clinical suspicion for a single unifying diagnosis, i.e., VEXAS syndrome. This patient presented with several aspects consistent with VEXAS syndrome, including an unexplained macrocytic anemia, elevated inflammatory markers, malaise, recurrent uveitis, venous thromboembolism (VTE), and evidence of immune dysfunction. Additionally, his age > 50 years and male sex fit the general demographic characteristics of patients with VEXAS syndrome. Prior to discharge, the patient underwent CT-guided bone marrow biopsy as well as genetic testing of peripheral blood for UBA1 gene mutation.

Case conclusion

After discharge, several important diagnostic test results were received. UBA1 mutation testing noted c.122 T>C; p.Met41Thr with a variant allele frequency of 94.1%. On bone marrow biopsy, the marrow was hypercellular with myeloid-predominant trilineage hematopoiesis and dysmegakaryopoiesis, without a significant increase in blasts (Figure 4). It did not demonstrate definitive morphologic or immunophenotypic evidence of HLH; however, occasional vacuolated myeloid precursors were noted (Figure 5). In addition, flow cytometry revealed no abnormal B- or T-cell populations, and stainable iron was adequate without ring sideroblasts, supporting overall a pattern of marrow dysplasia. Given these findings, the patient was ultimately diagnosed with VEXAS syndrome. Additionally, *Legionella* DNA PCR from pleural fluid returned positive, and his outpatient amoxicillin-clavulanate was changed to levofloxacin.

**Figure 4:**
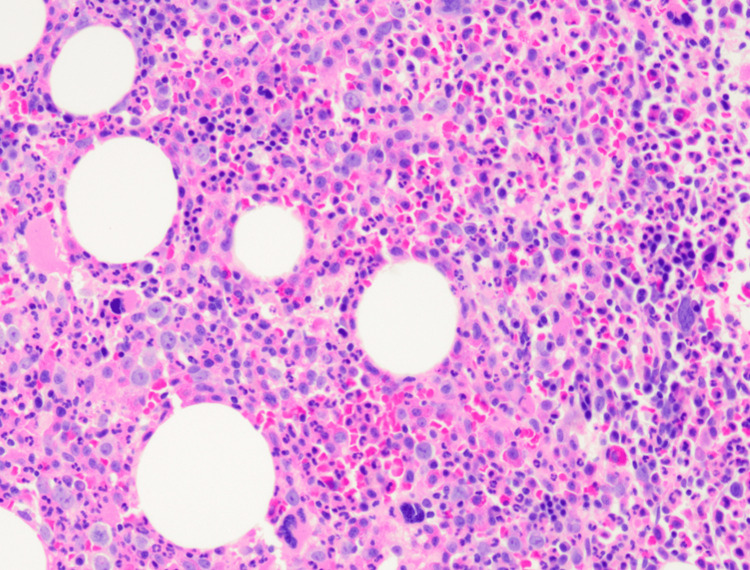
Bone marrow biopsy (H&E stain, high power). The bone marrow is hypercellular with myeloid-predominant trilineage hematopoiesis. Numerous maturing myeloid and erythroid precursors are present within a markedly cellular marrow space with reduced adipose tissue. Dysplastic changes were identified, particularly involving the megakaryocytic lineage, with dysmegakaryopoiesis characterized by occasional atypical small megakaryocytes. No significant increase in blasts is present.

**Figure 5:**
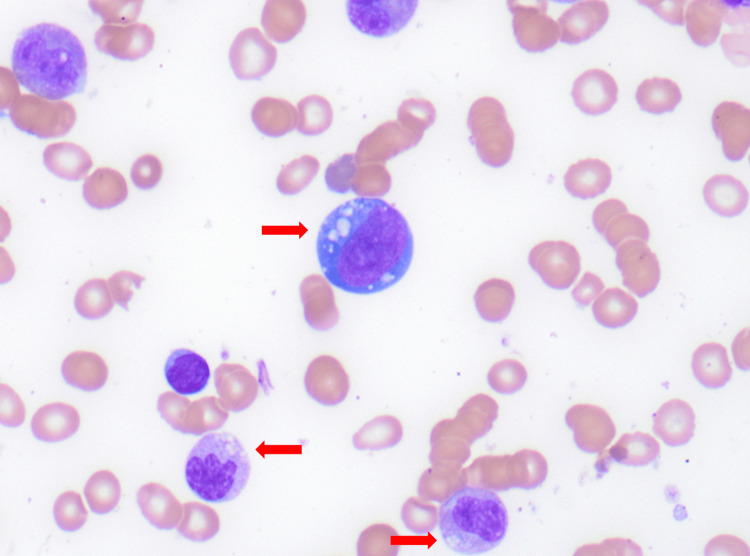
Bone marrow aspirate demonstrating vacuolization of myeloid precursor cells (red arrows).

Five days after discharge, the patient met with his outpatient hematologist and was started on prednisone 40 mg daily and trimethoprim-sulfamethoxazole prophylaxis with plans to taper prednisone as tolerated. On follow-up visits approximately two weeks after discharge, the patient noted feeling significantly better while on steroids. One month after discharge, he was started on azacitidine 75 mg/m^2^ per day for seven days to be repeated every four weeks for further treatment; additionally, his prednisone was weaned down to 20 mg daily. This ultimately resulted in the resolution of the patient's thrombocytopenia and a significant improvement in his anemia (Table 5).

**Table 5:** Trend in blood counts and inflammatory markers over time. PTA = prior to admission; PCP = primary care physician; Hgb = hemoglobin; MCV = mean corpuscular volume; WBC = white blood cells; ESR = erythrocyte sedimentation rate; CRP = C-reactive protein; H = high; L = low. * Reference ranges differ by performing laboratory. The values provided are the reference ranges of the admitting hospital laboratory.

Parameter	Initial rheumatology evaluation (6 months PTA)	Outpatient PCP visit (1 month PTA)	Day of admission	1 month post-discharge	3 months post-discharge	Reference range*
Treatment	Prednisone 20 mg daily	None	None	Prednisone 30 mg daily	Prednisone 20 mg daily + azacitidine	N/A
Hgb	14.2 g/dL	8.5 g/dL (L)	8.5 g/dL (L)	10.5 g/dL (L)	13.5 g/dL (L)	13.7-17.5 g/dL
MCV	107.4 fL (H)	113.9 fL (H)	112.9 fL (H)	122.3 fL (H)	119.9 fL (H)	79-94.8 fL
WBC	8.45 x 10^3^/µL	7.6 x 10^3^/µL	7.54 x 10^3^/µL	9.81 x 10^3^/µL	5.47 x 10^3^/µL	3.98-10.04 x 10^3^/µL
Platelets	102 x 10^3^/µL (L)	122 x 10^3^/µL (L)	91 x 10^3^/µL (L)	83 x 10^3^/µL (L)	236 x 10^3^/µL	163-369 x 10^3^/µL
ESR	9 mm/hr	128 mm/hr (H)	74 mm/hr (H)	8 mm/hr	–	≤12 mm/hr
CRP	0.3 mg/dL	11.7 mg/dL (H)	14.9 mg/dL (H)	0.6 mg/dL	–	<0.8 mg/dL
Ferritin	366 (H) ng/mL	–	1533 ng/mL (H)	–	–	8-350 ng/mL

## Discussion

This case highlights several important aspects of VEXAS syndrome, including classic manifestations, the need for increased provider awareness, potential complications of this syndrome, and the paucity of therapeutic options for these patients.

First, this case underscores a need for increased awareness of VEXAS syndrome, particularly for patients presenting with a cryptic autoinflammatory disease who meet the general demographic characteristics for patients with this condition. A diagnosis of VEXAS syndrome should be strongly considered in men over 50 years of age presenting with recurrent fevers, arthritis, rashes, or other inflammatory syndromes and hematologic abnormalities such as macrocytic anemia and thrombocytopenia. The diagnosis should be considered concurrently while the patient is being worked up for other diagnoses, given the overlap between this syndrome and others. If appropriate, diagnosis can be made via genetic testing for mutations in the UBA1 gene. Other findings that strongly support the diagnosis of VEXAS syndrome include vacuolization on bone marrow biopsy and neutrophilic dermatoses on skin biopsy [6]. This patient’s bone marrow also demonstrated hypercellularity, which is often seen in VEXAS patients [7], and dysmegakaryopoiesis, which is seen in both myelodysplastic syndromes and VEXAS syndrome. Additionally, this patient had a history of venous thromboembolism with a new pulmonary embolism identified during the hospitalization. VEXAS syndrome is associated with an increased risk for thrombosis, with one study noting VTE in over 40% of patients with VEXAS syndrome, many of whom were unprovoked [8]. Increasing clinician awareness of this disease’s manifestations may help expedite appropriate diagnostics and treatment.

Interestingly, the specific UBA1 mutation detected may also provide prognostic value. There are three canonical mutations in the UBA1 gene at the Met41 translation start site: p.Met41Val, p.Met41Thr, and p.Met41Leu. These mutations reduce the expression level of the UBA1 enzyme, which then leads to a decrease in levels of cellular ubiquitination. This is the mechanism that drives the various symptoms of VEXAS. Disease severity inversely correlates with the amount of UBA1 produced. For example, p.Met41Val results in the least amount of UBA1 production and is associated with the most severe disease phenotype, such as higher rates of inflammatory episodes, more transfusion dependence, and decreased survival. This patient had a p.Met41Thr mutation that is considered more intermediate risk and has been associated with the development of deep vein thrombosis and thrombocytopenia. In contrast, the p.Met41Leu mutation results in relatively higher levels of UBA1 and is therefore associated with a less severe inflammatory phenotype and better prognosis, although rates of myelodysplastic syndrome may be higher in this population [9,10].

The severity of this patient’s pneumonia further substantiates evidence of immunocompromise and infection risk in patients with VEXAS syndrome. This patient presented with a significant pneumonia resulting in hypoxemic respiratory failure. Yet, despite this, his white blood cell counts never responded appropriately. A recent review found that infections, particularly pneumonias caused by opportunistic pathogens, are common in patients with VEXAS syndrome and often severe, if not fatal [11]. Additionally, VEXAS patients are often treated with immunosuppressive medications, further increasing their risk of severe infection. It is worth noting, however, that this patient had many other risk factors for infection, including his age, diabetes, history of malignancy, and prior systemic corticosteroid use. Further investigation needs to be done on which infections these patients are at greatest risk for and whether they would benefit from the addition of antibiotic prophylaxis given their baseline immunocompromised state.

Unfortunately, there is no cure for VEXAS syndrome. Treatment often focuses on symptomatic management and reducing inflammasome activation, typically via glucocorticoid therapy. Other proposed therapeutic agents include tocilizumab, ruxolitinib, and azacitidine; however, most of the evidence supporting their use in VEXAS patients is limited to retrospective studies [12,13]. Alternatively, a recent study suggests that bone marrow transplant may be a promising treatment option in the future [14]. More research needs to be done to investigate the best treatment options for patients with VEXAS, as current options are limited and merely palliative in goal.

In this case, the patient was initially treated with prednisone and eventually started therapy with azacitidine. Azacitidine has been shown to be an effective disease-modifying therapy for VEXAS syndrome because it can address inflammation, cytopenias, and the UBA1 clonal burden simultaneously. In a retrospective French study of 88 VEXAS patients, almost half of the patients achieved near eradication of the UBA1 clone, which is in contrast to other VEXAS therapies that target cytokines and reduce symptoms but do not address the underlying mutant clone [15]. Interestingly, in this same study, there was also evidence that azacitidine improved inflammatory symptoms in patients who did not have evidence of myelodysplastic neoplasms, suggesting an indication for this patient without much dysplasia noted in his bone marrow biopsy. Given his older age and the recurrence of inflammatory flares when prednisone was tapered, he demonstrated a need for more definitive therapy that could be both steroid-sparing and disease-modifying.

## Conclusions

VEXAS syndrome can be an elusive diagnosis, despite it being a much more common entity than one might anticipate. The diagnosis should be strongly considered in patients presenting with an unexplained macrocytic anemia and recurrent signs and symptoms of inflammation, such as chondritis, uveitis, and skin rashes, especially those who are male and over 50 years of age. The signs, symptoms, laboratory abnormalities, and histologic findings may overlap considerably with other disorders, including vasculitis syndromes such as giant cell arteritis. The diagnosis is strongly supported by bone marrow biopsy demonstrating vacuolization of myeloid precursor cells and can be confirmed through genetic testing for UBA1 gene mutations.
